# Prolactin acts as a potent survival factor for human breast cancer cell lines

**DOI:** 10.1038/sj.bjc.6601947

**Published:** 2004-06-22

**Authors:** C M Perks, A J Keith, K L Goodhew, P B Savage, Z E Winters, J M P Holly

**Affiliations:** 1 Department of CSSB, Division of Surgery, Level 7, Bristol Royal Infirmary, Bristol BS2 8HW, UK

**Keywords:** breast cancer, prolactin, apoptosis

## Abstract

Human breast cancer is the leading cause of cancer death in women from Western societies, and a large study of the epidemiology demonstrated strong associations between human prolactin and risk of breast cancer. Using established models of apoptosis of human breast cancer cell lines, we assessed the role of prolactin in breast cancer cell growth and survival. We showed that prolactin had no effect on the metabolic activity or total cell number of any cell lines. We confirmed endogenous prolactin production by these cells and that the levels varied. In the presence of a prolactin-neutralising antibody, each of the cell lines responded with the induction of apoptosis as opposed to growth inhibition. The sensitivity of the cell lines to the physiological inducer of apoptosis, C2-ceramide, appeared relative to the levels of endogenous prolactin that they contained. We then showed that exogenously added prolactin acted as a potent survival factor against apoptosis in all the cell lines examined. In addition, we demonstrated that a prolactin-neutralising antibody in combination with C2-ceramide caused an anticipated, additive increase in cell death. This study demonstrated that prolactin protects human breast cancer cell lines against apoptosis and this may have important implications for cancer treatment.

Prolactin is a 23 kDa peptide hormone released from the lactotroph cell populations of the anterior pituitary gland. Prolactin has a well established role in stimulating breast growth and differentiation in puberty as well as lactation during pregnancy (Binart *et al*, 2000). Prolactin binds with its cell-surface receptor, which dimerises on prolactin binding triggering intracellular signalling (Goffin and Kelly, 1997). A hormone such as prolactin, whose normal action is to promote cell proliferation and differentiation, was soon identified as a potential candidate for the progression of breast cancer, where cells are proliferating and surviving inappropriately (Clevenger *et al*, 2003).

Indeed, the role of prolactin in rodent mammary cancer soon became clear. Transgenic female mice overexpressing the rat prolactin gene spontaneously developed mammary carcinomas (Rose-Hellekant *et al*, 2003). Conversely, drug-induced hypoprolactinaemia retarded tumour growth (Welsch *et al*, 1979).

Studies of the epidemiology have found that high serum prolactin levels were associated with known breast cancer risk factors such as parity status and mammographic breast density (Wang, 1988; Ingram *et al*, 1990). In addition, a prospective epidemiological study by Hankinson *et al* found strong evidence indicating that high serum prolactin levels were a risk factor for breast cancer in postmenopausal women (Hankinson *et al*, 1999).

In the human, clinical trials were undertaken with the aim of reducing serum prolactin levels using dopamine agonists such as bromocriptine (*in vivo*, dopamine inhibits prolactin release from the anterior pituitary gland). Although circulating prolactin levels were dramatically reduced, no therapeutic benefit in the breast cancer patients was seen (Bonneterre *et al*, 1988; Anderson *et al*, 1993). The failure of these clinical trials resulted in a diminished interest in prolactin as a therapeutic target in human breast cancer.

However, studies began to accumulate indicating that patients with surgical ablation of the anterior pituitary gland still had detectable levels of prolactin (Lachelin *et al*, 1977), which suggested the existence of extrapituitary sites of prolactin production. Indeed, several laboratories have since demonstrated the synthesis of prolactin in breast cancer cells and normal breast tissue, raising the possibility that prolactin may act in an autocrine/paracrine manner within the mammary gland (Fields *et al*, 1993). mRNA for prolactin and its receptor has been found in normal breast tissues and in primary human breast cancers; while both receptor mRNA and protein are expressed in nearly all human breast cancers, they are not generally overexpressed (Mertani *et al*, 1998) (Reynolds *et al*, 1997). Overexpression of the receptor mRNA has been demonstrated in some breast cancer cell lines (Peirce and Chen, 2001). Recent studies showing inhibition of cell growth and survival of breast cancer cells in the presence of prolactin blocking antibodies and receptor antagonists also support an autocrine/paracrine loop of locally produced prolactin (Ginsburg and Vonderhaar, 1995; Ramamoorthy *et al*, 2001). These data provide an explanation for the failure of the dopamine agonist trials, since lowering prolactin release from the pituitary gland would have had no effect on the proliferation of breast cancer cells initiated by a local source of prolactin.

Administration of most chemotherapeutic agents, including those used for treatment of breast cancer, eventually leads to the onset of programmed cell death or apoptosis. The actions of these anticancer drugs on apoptosis are primarily mediated by the induction of endogenous ceramide (Ogretmen and Hannun, 2001). It is clear that ceramide plays an important role in the response of cancer cells to chemotherapeutic drugs. We have previously established inducible models of apoptosis in breast cancer epithelial cell lines using an analogue of ceramide, C2-ceramide, as a trigger of cell death (Gill *et al*, 1997; Perks *et al*, 1999). The aims of this study were to use these models to assess the role of prolactin in breast cancer cell growth and survival with a view to increasing our understanding of its obvious potential as a therapeutic target.

## MATERIALS AND METHODS

### Materials

Prolactin peptide was purchased from the National Hormone and Peptide Programme (Dr AF Parlow) and prolactin peptide purified from human pituitary glands was bought from Sigma, Poole, Dorset, UK. These two sources of prolactin peptide were compared and found to be equally potent. IGF-I peptide was purchased from GroPep Ltd, Adelaide, Australia. C2-ceramide and EGF peptide were purchased from Calbiochem, Nottingham, UK. The prolactin monoclonal antibody was bought from QED Bioscience Inc., San Diego, CA, USA and the control mouse IgG antibody was obtained from DAKO, Denmark. All other materials were obtained from Sigma Poole, Dorset, UK.

### Cell culture

Human breast cancer cell lines MDA-MB-231 (EGF responsive, IGF-I nonresponsive), T47D (EGF responsive, IGF-I nonresponsive), MCF-7 (IGF-I responsive, EGF nonresponsive) and Hs578T (IGF-I nonresponsive, EGF responsive) cells were purchased from the American Type Culture Collection (ATCC) and grown in a humidified 5% CO_2_ atmosphere at 37°C. MDA-MB-231, T47D, MCF-7 and Hs578T cells were maintained in Dulbecco's modified Eagle's medium (DMEM) supplemented with 10% foetal calf serum, penicillin (50 IU ml^−1^), streptomycin (50 *μ*g ml^−1^) and L-glutamine (2 mM) growth media (GM). Experiments for all cell lines were performed in phenol red- and serum-free HEPES DMEM and Ham's nutrient mix F-12 (SFM) with sodium bicarbonate (0.12%), bovine serum albumin (0.2 mg ml^−1^), transferrin (0.01 mg ml^−1^) and supplemented with antibiotics as before (SFM).

### Dosing protocol

Cells were grown in GM for 24 h before switching to SFM for a further 24 h, prior to dosing for a further (a) 48 h with either prolactin (0–100 ng ml^−1^), IGF-I (20 ng ml^−1^) or EGF (0–100 ng ml^−1^) and (b) 24 h with either a prolactin blocking antibody (100 ng ml^−1^), a control mouse IgG (100 ng ml^−1^), prolactin (0–100 ng ml^−1^) or C2-ceramide (0–50 *μ*M) alone. Cells were also treated with an apoptotic dose of C2-ceramide in combination with either a prolactin blocking antibody (100 ng ml^−1^), a control mouse IgG (100 ng ml^−1^) or with prolactin (100 ng ml^−1^). The dose of C2-ceramide was chosen to achieve approximately 50% cell death, which varied from 20–50 *μ*M depending upon cell type and passage. We have shown previously that C2-ceramide induces apoptosis in all of the above cell lines and that levels of cell death measured by Trypan blue cell counting correlate with levels of apoptotic cells measured by flow cytometry in these models (Gill *et al*, 1997; Perks *et al*, 1999).

### Trypan blue dye exclusion

Aliquots of cell suspension were loaded onto a haemocytometer (1 : 1) with Trypan blue dye. Viable cells exclude the dye. Both living and dead cells were counted (total cell number) from which the percentage of dead cells relative to control was calculated.

### 3-(4,5-Dimethylthiazol-2-yl-2,5-diphenylterazolium bromide) (MTT) Assay

Cells were seeded at 2.5 × 10^4^ ml^−1^ (150 *μ*l GM) in 96-well plates and were allowed to grow for 24 h. Growth medium was replaced with SFM 24 h before dosing. 3-(4,5-Dimethylthiazol-2-yl-2,5-diphenylterazolium bromide reagent (7.5 mg ml^−1^) in phosphate-buffered saline was added to the cells (10 *μ*l well^−1^) and the cultures were incubated for 30 min at 37°C. The reaction was stopped by the addition of acidified triton buffer (0.1 M HCl, 10% (v v^−1^) Triton X-100; 50 *μ*l well^−1^); tetrazolium crystals were dissolved by mixing on a Titertek plate shaker for 20 min at room temperature. The samples were measured on a Bio-Rad 450 plate reader at test wavelength of 595 nm and a reference wavelength of 650 nm.

### Western immunoblotting

Cells (1 × 10^6^) were grown to 70% confluency in GM, which was replaced with SFM for 24 h. Cells were then lysed on ice for 10 min (1 ml; 10 mM Tris-HCl, 5 mM EDTA, 50 mM NaCl, 30 mM Na pyrophosphate, 50 mM sodium fluoride, 100 *μ*M sodium orthovanadate, 1% Triton, 1 mM phenylmethylsulphonyl fluoride; pH 7.6). Normalised amounts of proteins were loaded and separated by 12.5% sodium dodecyl sulphate–polyacrylamide gel electrophoresis and then transferred onto a nylon membrane. Nonspecific binding sites were blocked (5% milk in TBST) and the membrane was then probed with antiprolactin (1 *μ*g ml^−1^) overnight. Following the removal of excess unbound antibody, an anti-mouse antibody conjugated to peroxidase (1 : 2000) was added for 1 h. Binding of the peroxidase was visualised by enhanced chemiluminescence according to the manufacturer's instructions. Optical density measurements were determined using a scanning densitometer (Biorad, Hemel Hempstead, UK) and analysed using Molecular Analyst software (Biorad, Hemel Hempstead, UK). The protein content of each sample was determined using a BCA Protein Assay Reagent Kit.

### Statistical analysis

The data were analysed using the Microsoft Excel 97 version 4.0 software package. Significant effects were determined using ANOVA followed by Student's *t*-test. A statistically significant difference was considered to be present at *P*<0.05.

## RESULTS

### Effects of prolactin on the proliferation of breast cancer cells

**Figure 1:**
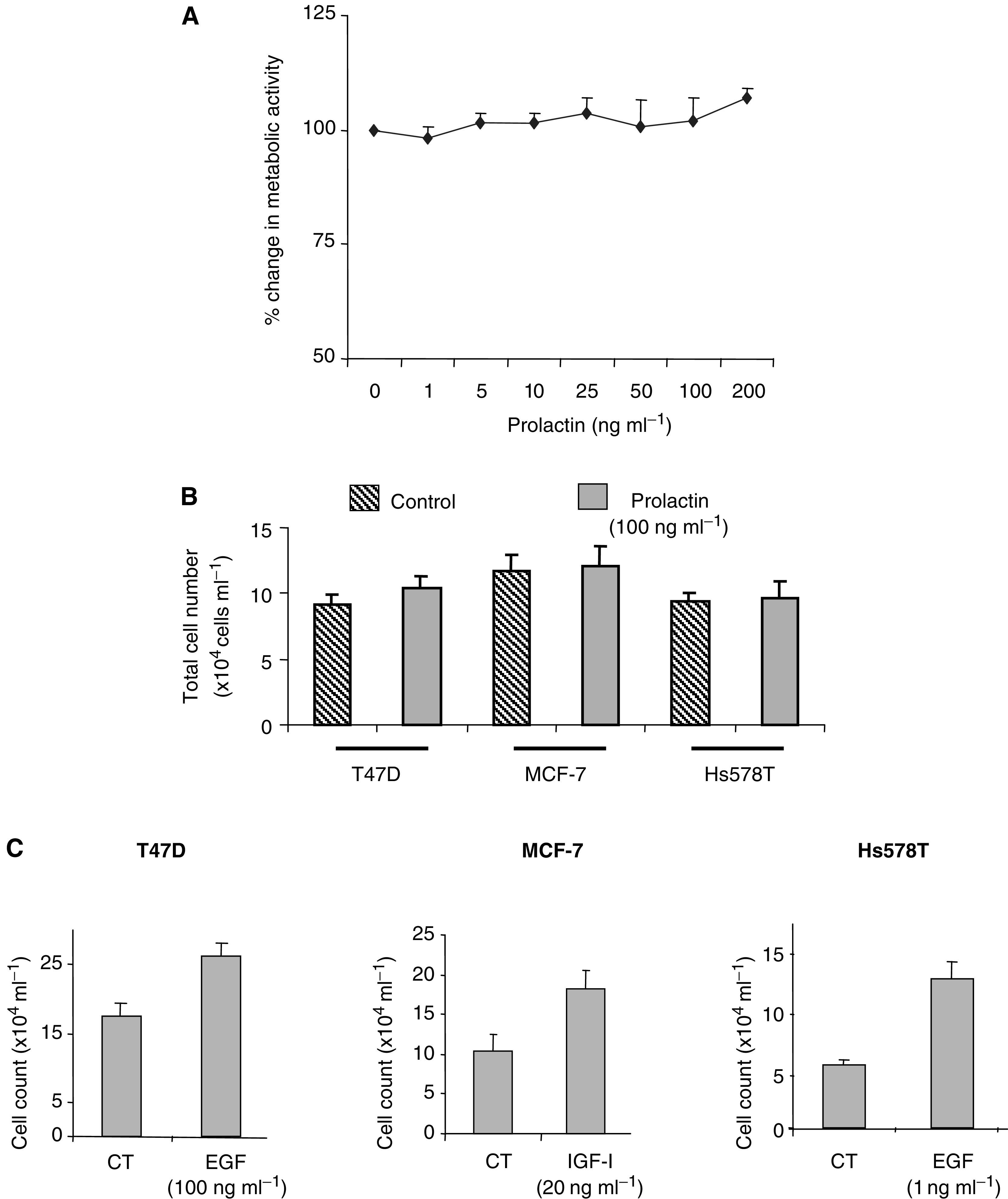
Effects of prolactin on the proliferation of breast cancer cells. Effects of prolactin (0–200 ng ml^−1^) for 48 h on (**A**) metabolic activity of T47D human breast cancer cells and (**B**) total cell counts in T47D, MCF-7 and Hs578T human breast cancer cells. (**C**) Total cell counts after treatment for 48 h with EGF (1 and 100 ng ml^−1^) in Hs578T and T47D cells, respectively, and of IGF-I (20 ng ml^−1^) in MCF-7 cells. Graphs show experiments performed in triplicate, which are repeated at least three times.

Prolactin (1–200 ng ml^−1^) had no effect on the metabolic activity of T47D cells (Figure 1A

) over a 48 h period. Similar dose responses were performed in the MCF-7 and Hs578T cell lines and no effects on metabolic activity were observed (data not shown). Using prolactin at 100 ng ml^−1^, we then confirmed in each of the above cell lines that there was no significant increase in total cell number (Figure 1B). Despite observing no effect of prolactin on cell proliferation, we did observe increases in cell growth in these cell lines (ranging from 1.4- to two-fold) over 48 h with either EGF or IGF-I (Figure 1C).

### Endogenous prolactin production correlates to sensitivity to apoptosis

**Figure 2:**
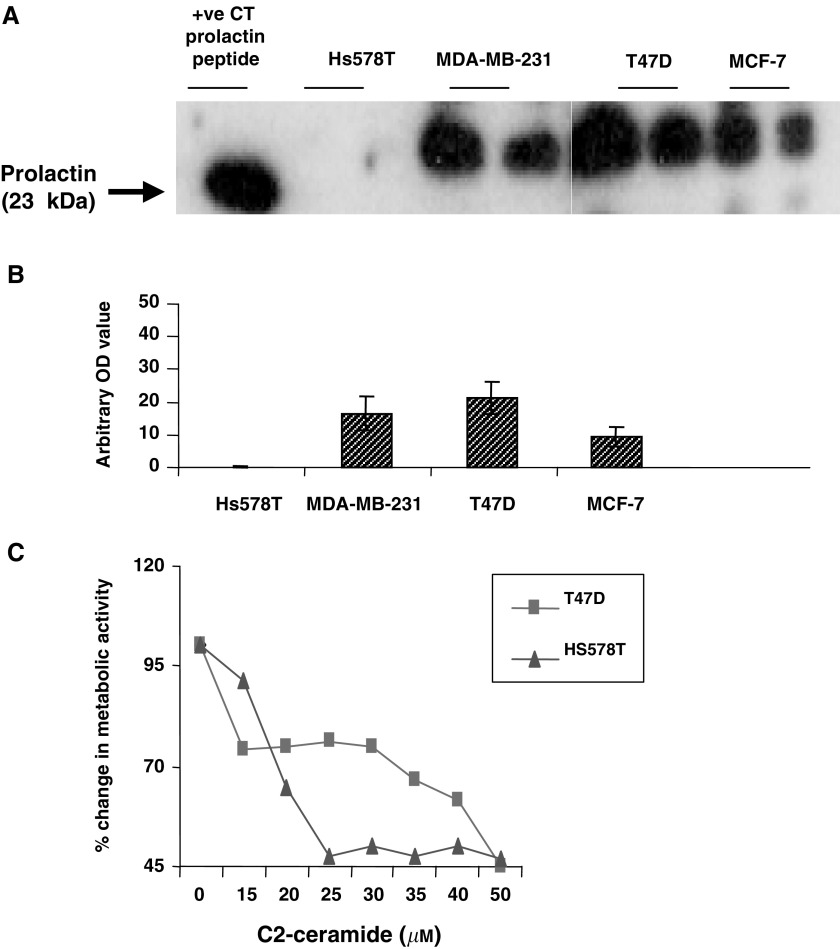
Endogenous prolactin production correlates to sensitivity of breast cancer cells to apoptosis. (**A**) Shows a Western immunoblot for prolactin in equal amounts of whole-cell lysates from Hs578T, MDA-MB-231, T47D and MCF-7 cells, where prolactin peptide is used as a positive control. (**B**) Demonstrates the arbitrary optical density measurements from Western immunoblots assessing prolactin levels. (**C**) Shows the percentage change in metabolic activity in response to C2-ceramide (0–50 *μ*M) treatment for 24 h in T47D and Hs578T cells. All experiments were repeated at least three times.

It has been conclusively demonstrated that human normal breast and breast cancer cell lines produce their own prolactin (Clevenger *et al*, 1995; Ginsburg and Vonderhaar, 1995). We confirmed that prolactin was present in the T47D, MCF-7, MDA-MB-231 and Hs578T breast cancer cell lines. The prolactin found in the cell lysates ran at a slightly higher molecular weight than the prolactin peptide used as a positive control. This could be due to prolactin post-translational modifications such as glycosylation or phosphorylation (Sinha, 1995; Bollengier *et al*, 2001; Gobello *et al*, 2001). The prolactin levels varied accordingly: highest in T47-D> MDA-MB-231> MCF-7>> lowest in Hs578T (Figure 2A

). Using the cell line with either the highest (T47-D) or lowest (Hs578T) level of prolactin, we examined the sensitivity (in terms of changes in metabolic activity) to the apoptotic trigger C2-ceramide. In response to C2-ceramide, we found that the T47-D cells were the least sensitive and the Hs578T cells were the most sensitive, and this appeared to correlate with their relative levels of endogenous prolactin (Figures 2B and C). For example, at 25 *μ*M C2-ceramide, there was only a 23.7% decrease in metabolic activity in the T47D cells in comparison to a 52.5% decrease in the Hs578T cells. We also performed Western immunoblotting with the U5 prolactin receptor from Alexis Biochemicals, Nottingham, UK, and found that all the cell lines possessed the 40 kDa short form of the receptor but to different degrees (Arbitrary OD units: MDA-MB-231=6.3; T47D=3.5; MCF-7=2.8; Hs578T=2.1). The relative levels of prolactin produced followed a similar order.

### Effects of a prolactin blocking antibody on apoptosis

**Figure 3:**
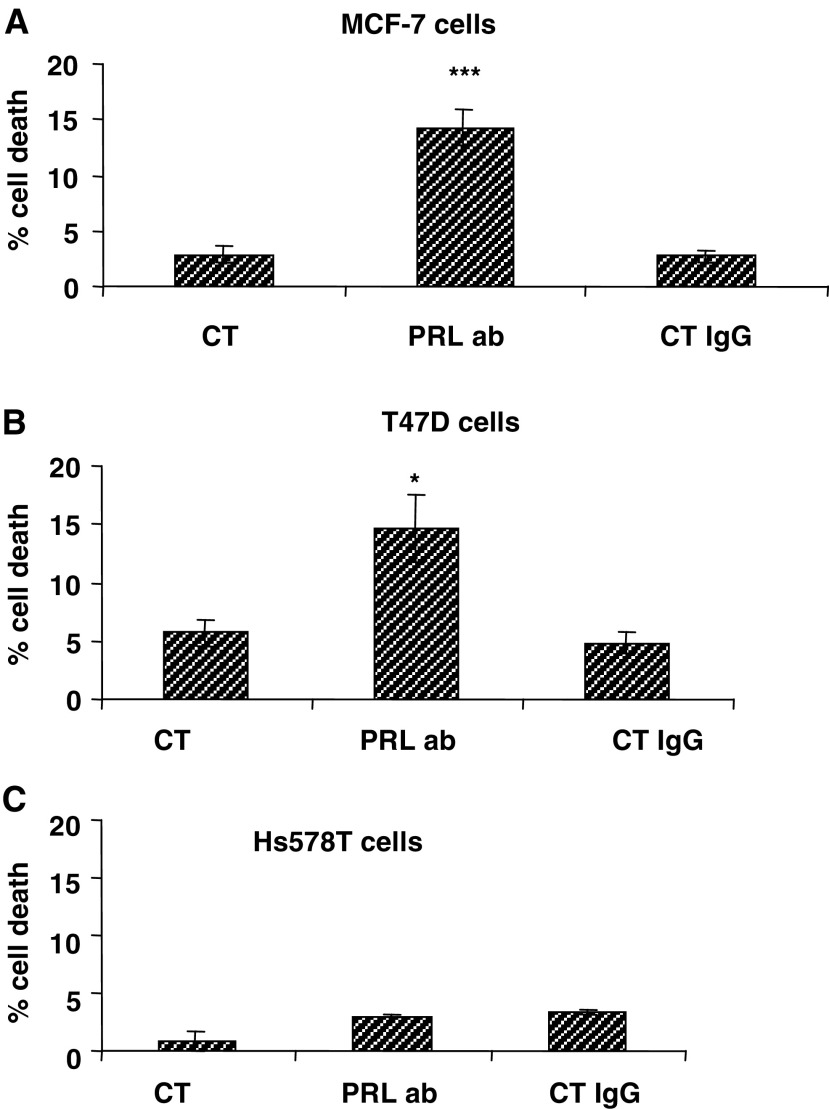
Effects of a prolactin-neutralising antibody on apoptosis. Cell death was measured in (**A**) MCF-7, (**B**) T47D and (**C**) Hs578T cells following treatment with either a prolactin blocking antibody (100 ng ml^−1^) or a control mouse IgG (100 ng ml^−1^) for 24 h. Graphs represent the mean of three experiments each performed in triplicate, where ^*^P<0.05 and ^***^P<0.01.

In the presence of a prolactin blocking antibody, there was a significant increase in cell death from 2.8 to 14.3% in the MCF-7 cells (*P*<0.001) (Figure 3A

) and from 5.7 to 14.5% in the T47D cells (*P*<0.05) (Figure 3B). Since there were negligible levels of endogenous prolactin in the Hs578T, as we anticipated there was no significant difference in the levels of cell death in the presence of the prolactin blocking antibody (Figure 3C). The control mouse IgG had no effect on cell death in any cell line.

### Effects of prolactin on C2-ceramide-induced apoptosis

Figure 4A

**Figure 4:**
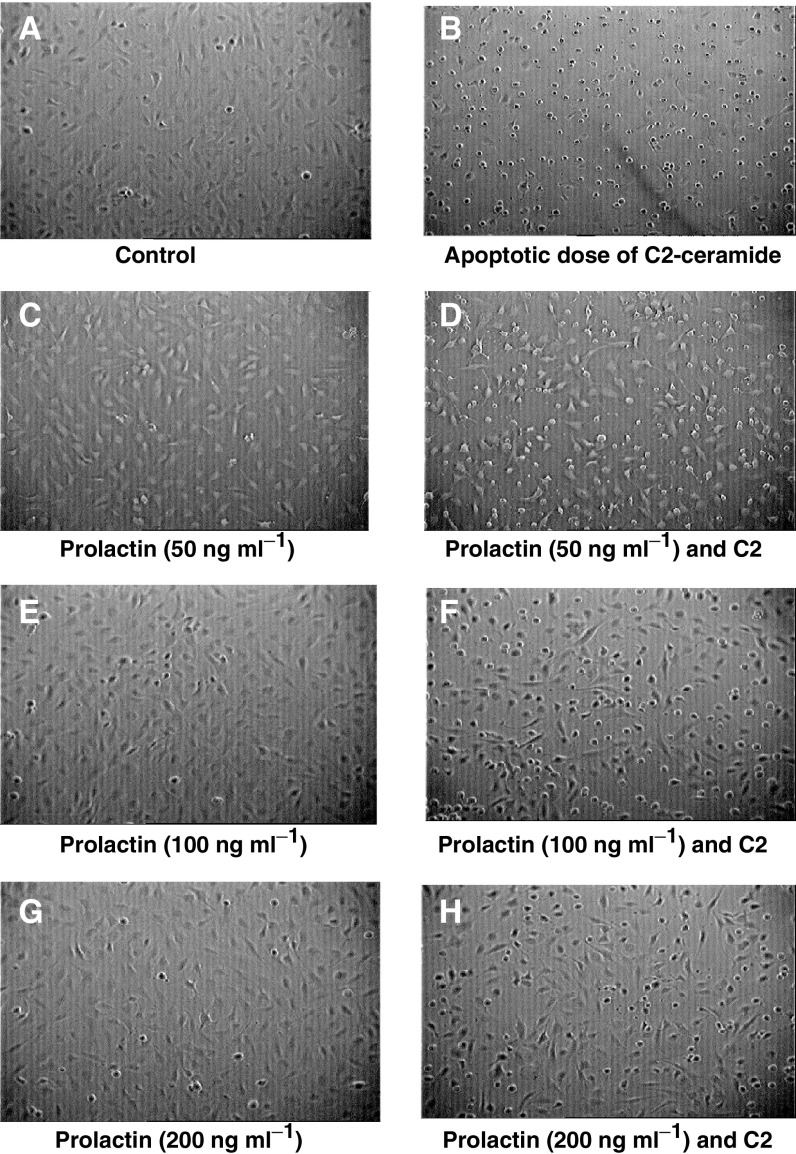
Photomicrographs to demonstrate that C2-induced apoptosis is dose dependently decreased by the addition of prolactin (50, 100 and 200 ng ml^−1^) in Hs578T cells. a=untreated cells; b=apoptotic dose of C2-ceramide; c=prolactin (50 ng ml^−1^); d=prolactin (50 ng ml^−1^ and C2); e=prolactin (100 ng ml^−1^); f=prolactin (100 ng ml^−1^ and C2); g=prolactin (200 ng ml^−1^); h=prolactin (200ng ml^−1^ and C2) (magnification × 100).

shows untreated control Hs578T cells. Figure 4C, E and G shows the addition of increasing doses of prolactin (50–200 ng ml^−1^), indicating no effect on the cells relative to controls. Figure 4B represents cells 24 h after treatment with an apoptotic dose of C2-ceramide. This illustrates distinct rounding of the cells and a reduction in the number of cells attached to the plate. Figure 4D, F and H show coincubation of C2 with increasing doses of prolactin (50, 100 and 200 ng ml^−1^, respectively). The number of rounded, dead cells is clearly dose dependently reduced by prolactin relative to C2 alone. We determined by cell counting that prolactin at 100 ng ml^−1^ reduced C2-induced cell death by approximately 30%, and so chose this dose of prolactin for all further experiments.

In Figures 5A–C

**Figure 5:**
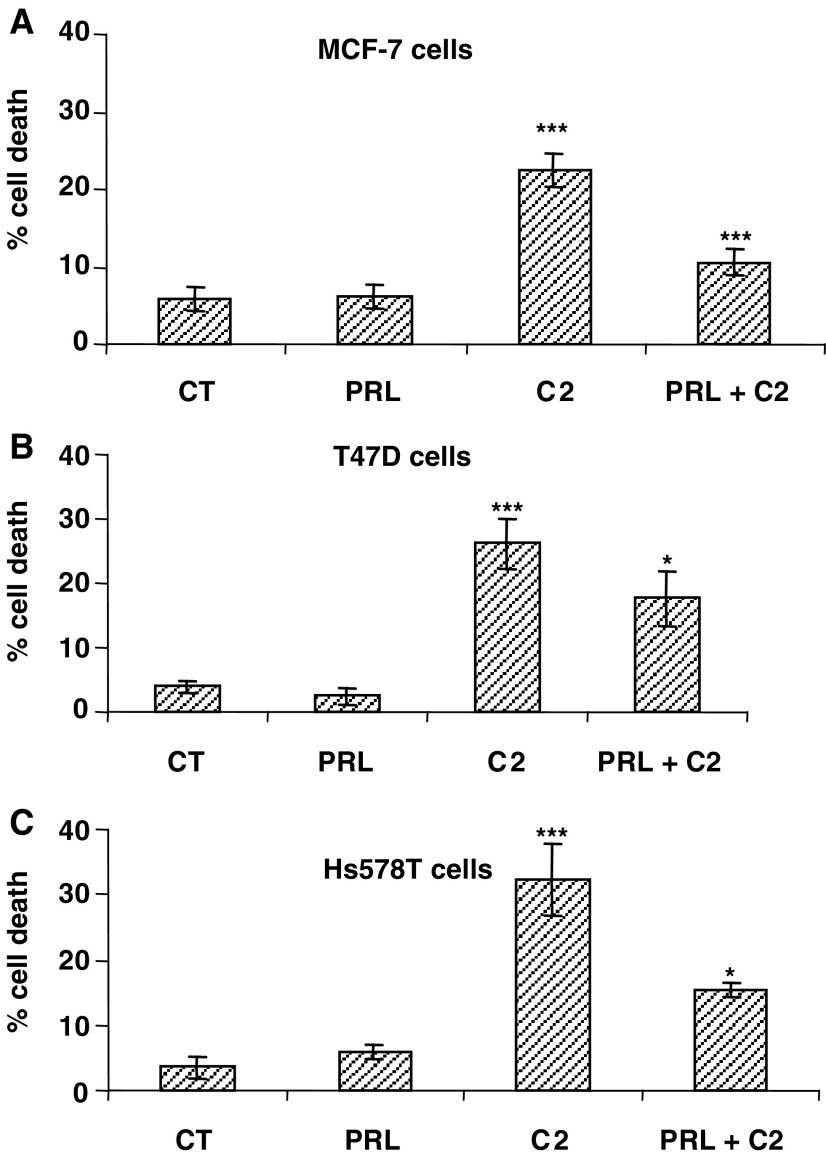
Effects of prolactin on C2-ceramide-induced apoptosis. Cell death was measured in (**A**) MCF-7, (**B**) T47D and (**C**) Hs578T cells following treatment with either prolactin (100 ng ml^−1^), an apoptotic dose of C2-ceramide or the combination of the two. Graphs represent the mean of three experiments each performed in triplicate, where ^*^P<0.05 and ^***^P<0.01.

, prolactin alone (100 ng ml^−1^) had no effect on basal levels of cell death in either the MCF-7, T47D or Hs578T cells. C2-ceramide induced significant levels of apoptosis from 5.8 to 22.4% in the MCF-7 cells (*P*<0.001), from 4.0 to 26.1% in the T47D cells (*P*<0.001) and from 3.5 to 32.2% in the Hs578T cells (*P*<0.001).

Prolactin in combination with C2-ceramide conferred significant cell survival in each case from 22.4 to 10.6% in the MCF-7 cells (*P*<0.001), from 26.1 to 17.7% in the T47D cells (*P*<0.05) and from 32.2 to 15.4% in the Hs578T cells (*P*<0.05). As anticipated due to the small amounts of endogenous prolactin, the degree of survival was greater in the Hs578T cells than in the T47D cells (57.9% reduction in death compared to 38.1%).

### Effects of C2-ceramide in combination with a prolactin blocking antibody

**Figure 6:**
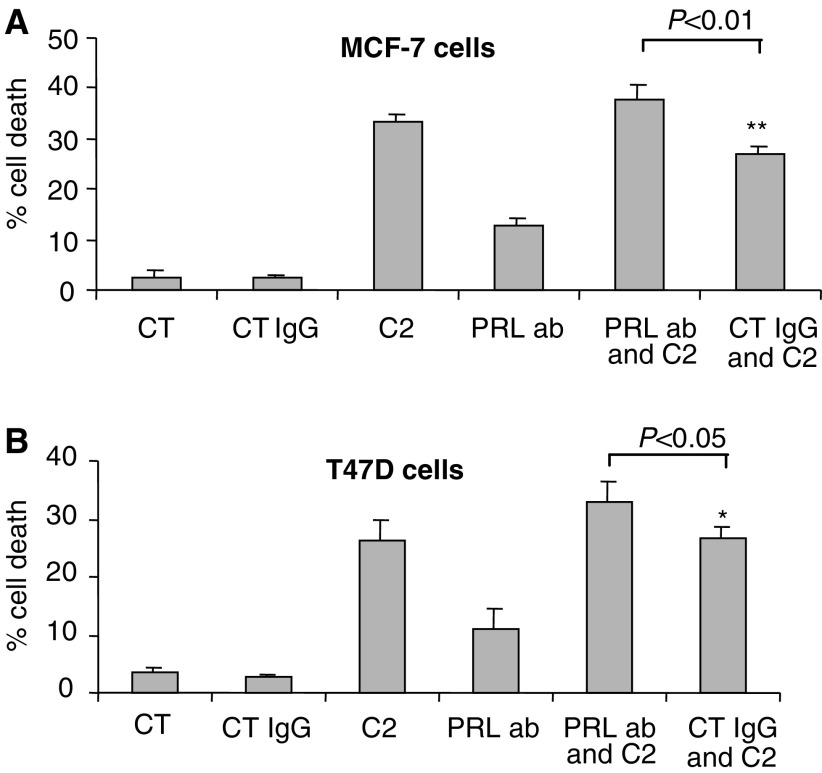
Effects of C2-ceramide in combination with a prolactin-neutralising antibody. Cell death was measured in (**A**) MCF-7 and (**B**) T47D cells following treatment with an apoptotic dose of C2-ceramide or a prolactin blocking antibody or a control mouse IgG or a combination of C2-ceramide with either a prolactin-neutralising antibody or a control mouse IgG. Graphs represent the mean of at least six replicate, where ^*^P<0.05 and ^**^P<0.01.

As shown previously in Figures 3A, B and 6A, B

, C2-ceramide and a prolactin blocking antibody each alone increased basal levels of cell death. In Figure 6A, B, as anticipated, we demonstrated a significant additive increase in apoptosis in the presence of C2-ceramide in combination with a prolactin blocking antibody in both the MCF-7 (*P*<0.01) (Figure 6A) and the T47D (*P*<0.05) (Figure 6B) cell lines relative to C2 in the presence of the control IgG. There were no differences in cell death between C2-ceramide and the combination of C2-ceramide and a control mouse IgG in either cell line.

## DISCUSSION

Human breast cancer is the leading cause of cancer death in women from Western societies, and a large study of the epidemiology has demonstrated strong associations between human prolactin and risk of breast cancer (Wang *et al*, 1988; Hankinson *et al*, 1999). Despite a number of studies showing that prolactin promotes cell proliferation in some breast cancer cell lines (Fuh and Wells, 1995), our investigations showed that prolactin had no effect on cell proliferation in any cell line over the 48 h tested. This suggested that the breast cancer cell lines must be producing their own prolactin as has been demonstrated previously (Fields *et al*, 1993). We confirmed that the breast cancer cells lines we were studying did contain prolactin and that the levels varied between lines. The T47D cells contained the highest levels, followed by MDA-MB-231 cells and MCF-7 cells and the lowest levels were found in the Hs578T cells. These findings are consistent with a previous study which measured levels of prolactin and found that T47D cells produced appreciably higher levels of prolactin in comparison to the MCF-7 and MDA-MB-231 cells (Ginsburg and Vonderhaar, 1995).

Whereas some studies have reported that neutralising prolactin antibodies caused a decrease in proliferation in MCF-7 and T47D cells (Ginsburg and Vonderhaar, 1995), we did not observe any such effects on cell growth (data not shown). However, we did find that the cells responded in the presence of a prolactin-neutralising antibody with the induction of apoptosis, which has also been observed by others (Chen *et al*, 1999). This suggested that the endogenous prolactin was not sufficient to drive proliferation, but was crucial for cell survival. In support of prolactin being a potent survival factor, we observed that the sensitivity of the breast cancer cell lines to the physiological inducer of apoptosis, C2-ceramide, appeared relative to the levels of endogenous prolactin that they contained. We determined that T47D cells (highest levels of prolactin) were more resistant to the induction of cell death by C2-ceramide than the Hs578T cells (lowest levels of prolactin). To confirm these observations, we then induced apoptosis and showed for the first time that exogenously added prolactin does act as a potent survival factor against C2-ceramide-induced apoptosis in breast cancer cell lines. In addition, we demonstrated that a prolactin-neutralising antibody in combination with C2-ceramide caused an anticipated, additive increase in cell death.

These data may have important implications for cancer treatment. A number of current cancer therapies are mediated via endogenous ceramide production (reviewed in Radin, 2003). Our data would suggest that endogenous levels of prolactin in breast tumours may be indicative of the efficacy of current treatment regimens designed to eliminate cancer cells via modulation of endogenous ceramide production. It may be that tumours with high levels of endogenous prolactin would respond poorly to such treatments, whereas those with lower levels may give a much better response. This is supported by a clinical study, which described how inhibiting prolactin secretion using agents such as bromocriptine enhanced the efficacy of chemotherapeutic drugs for the treatment of breast cancer (Lissoni *et al*, 2002). Therefore, tumours with high levels of prolactin would perhaps be more efficiently treated with additional antiprolactin/prolactin receptor therapies.

In summary, we have shown that prolactin acts as a potent survival factor for human breast cancer cell lines, which has also been demonstrated for Nb2 lymphoma cells (Fernandez *et al*, 2003), thymocytes (Krishnan *et al*, 2003) and the PC3 prostate cancer cell line (Ruffion *et al*, 2003). In addition, we found that the levels of endogenous prolactin made by the breast cancer cell lines appeared to correlate with their sensitivity to a physiological inducer of apoptosis, C2-ceramide. Our data showing that prolactin has the ability to prevent breast cancer cells from undergoing apoptosis, in addition to other reports indicating a role for prolactin in promoting cell motility (Maus *et al*, 1999) and angiogenesis (Struman *et al*, 1999) suggest that prolactin has the capacity to contribute significantly to the metastatic phenotype of breast cancer. Assessing prolactin concentrations within breast tumours may allow us to predict the response to current chemotherapeutic drugs; in addition, it supports the use of effective prolactin antagonists, since they may provide a better, more effective therapeutic intervention for some breast cancers.
